# Developing a Reliable Holographic Flow Cyto-Tomography Apparatus by Optimizing the Experimental Layout and Computational Processing

**DOI:** 10.3390/cells11162591

**Published:** 2022-08-19

**Authors:** Jaromír Běhal, Francesca Borrelli, Martina Mugnano, Vittorio Bianco, Amedeo Capozzoli, Claudio Curcio, Angelo Liseno, Lisa Miccio, Pasquale Memmolo, Pietro Ferraro

**Affiliations:** 1 Institute of Applied Sciences and Intelligent Systems, Italian National Research Council (CNR-ISASI), 80078 Pozzuoli, Italy; 2 Dipartimento di Ingegneria Elettrica e delle Tecnologie dell’Informazione, Università di Napoli Federico II, 80125 Napoli, Italy

**Keywords:** holographic microscopy, phase-contrast tomography, single-cells analysis

## Abstract

Digital Holographic Tomography (DHT) has recently been established as a means of retrieving the 3D refractive index mapping of single cells. To make DHT a viable system, it is necessary to develop a reliable and robust holographic apparatus in order that such technology can be utilized outside of specialized optics laboratories and operated in the in-flow modality. In this paper, we propose a quasi-common-path lateral-shearing holographic optical set-up to be used, for the first time, for DHT in a flow-cytometer modality. The proposed solution is able to withstand environmental vibrations that can severely affect the interference process. Furthermore, we have scaled down the system while ensuring that a full 360° rotation of the cells occurs in the field-of-view, in order to retrieve 3D phase-contrast tomograms of single cells flowing along a microfluidic channel. This was achieved by setting the camera sensor at 45° with respect to the microfluidic direction. Additional optimizations were made to the computational elements to ensure the reliable retrieval of 3D refractive index distributions by demonstrating an effective method of tomographic reconstruction, based on high-order total variation. The results were first demonstrated using realistic 3D numerical phantom cells to assess the performance of the proposed high-order total variation method in comparison with the gold-standard algorithm for tomographic reconstructions: namely, filtered back projection. Then, the proposed DHT system and the processing pipeline were experimentally validated for monocytes and mouse embryonic fibroblast NIH-3T3 cells lines. Moreover, the repeatability of these tomographic measurements was also investigated by recording the same cell multiple times and quantifying the ability to provide reliable and comparable tomographic reconstructions, as confirmed by a correlation coefficient greater than 95%. The reported results represent various steps forward in several key aspects of in-flow DHT, thus paving the way for its use in real-world applications.

## 1. Introduction

Nowadays, the in-flow analysis of cellular populations is a fundamental step for a whole host of technologies, ranging from well-established clinical trials to new tools that are currently being developed. The gold standard in this field is the commercially available Fluorescence-Activated Cell Sorter (FACS), which is a high-throughput flow cytometer able to sort cell subpopulations with high efficiency [1]. FACS has high specificity, which is based on the combined detection of morphological differences mainly obtained through fluorescence labeling. The main drawbacks of marker-based technologies arise from the a-priori knowledge of the targets to be labeled, the several biological protocols needed for sample preparation, and the massive use of chemical reagents. Moreover, the invasive and destructive nature of staining processes constitutes a significant motivation for researchers to develop label-free tools. Innovative no-contact and marker-free strategies have been modeled and tested in microfluidics and optics [2,3,4,5,6], and are set to become the key technologies in the biomedical field. Among them, Quantitative Phase Imaging (QPI) is a promising technique because the retrieved images quantitatively measure the optical thickness [7,8]. QPI technologies were first developed on adherent samples [9,10]. Nevertheless, in the last few years, the possibility of combining QPI with microfluidic strategies for high-throughput screening has started to be investigated [11,12,13,14]. Basic QPI techniques retrieve the integrated phase along the light-propagation direction, thus neglecting the 3D sample structure. Deep and detailed insight into single-cell anatomy is feasible if methods of phase-contrast tomography are incorporated, in order to exploit angular-dependent observations of the studied specimen. In particular, Digital Holographic Tomography (DHT) provides the 3D refractive index (RI) distribution of the investigated sample, as is encoded within the measured optical path difference maps. Data processed in DHT consist of multiple angular-dependent acquisitions of the sample, with the scanning realized by a variable oblique illumination, a sample rotation, or a combination of both approaches, leading to improved resolution isotropy [15]. Standard experimental realizations of DHT are mostly based on modifications of the Mach–Zehnder interferometer architecture [16,17,18,19,20,21]. In the case of the static illumination beam, the sample rotation approach is commonly assumed. Recently, a completely new step forward has been demonstrated for obtaining phase-contrast tomography images in a flow-cytometry modality by having cells flowing along microfluidic channels [22,23,24,25,26]. The flowing cells can experience self-rotation, thanks to the shear flow or rolling on the channel-side wall [24], allowing the 3D phase-contrast tomography of every flowing cell within the field of view (FoV). This concept simplifies tomographic microscopes to a remarkable degree, as the cell is probed along multiple directions as it passes through the FoV. The optical arrangement assumed in [26] is based on a Mach–Zehnder interferometer. The main challenge is faces concerns the implementation of a robust strategy for the rotation angle recovery [27] in order to apply the propagation algorithms for 3D tomographic imaging, which is the slice-by-slice distribution of the RI inside the volume of each flowing cell. Even if the target has been successfully achieved in recent years, in-flow phase-contrast tomography requires a bulky setup, intended for use in optical laboratories with highly skilled personnel; the main hardware equipment is based on an oil-immersion 40x objective with a high numerical aperture (i.e., NA = 1.3) and a 5120 × 5120 pixel camera for large FoV inspection [26], thus guaranteeing both high-resolution and high-throughput imaging. Moreover, the main disadvantage of Mach–Zehnder based optical systems concerns the independent optical paths of the mutually coherent signal and reference waves that are subsequently mixed to create the interference record. This approach exhibits high sensitivity to mechanical vibrations because both separated waves face uncorrelated disturbances, causing additional amplitude-phase noise during the recording of the interference pattern (i.e., the digital hologram). The loss of temporal stability can be overcome by incorporating common-path approaches, where interfering waves share identical or similar optical paths [28,29,30], thus compensating for environmental vibrations. In common-path configurations, increased attention has to be paid to designing a uniform reference wave, allowing for the primary classification of common-path approaches [31,32,33,34,35] as point diffraction or lateral shearing, which are appropriate for compact optical systems. The Shearing Device (SD) is incorporated in lateral shearing approaches, introducing the spatial carrier frequency and duplicating the FoV. A portion of the observed area of the sample acts as a reference wave, thus just part of the FoV is exploited, making these methods suitable for imaging spatially sparse and bounded samples. Various SDs have been implemented, including a beam splitter with mirrors in Michelson [36,37] or Sagnac [38] geometry, a thick glass plate [39,40,41], a specially oriented beam splitter [42], a Wollaston prism [43], a beam displacer [44], a Rochon polarizer [29], a Fresnel biprism [45], and diffraction grating [46]; here, the critical step was resolved, allowing the shearing distance to be adjusted independently on the interference fringes. Here, we show a comprehensive approach for optimizing various critical issues of a DHT system in combination with a microfluidic cytometer. In order to achieve a more compact and easy-to-use device, the key issues are a long-working-distance objective, and a camera with a relatively small sensor area and a lower recording frame rate than that of the previous bulk configurations [26]. For the first time, we designed and tested an experimental layout based on a quasi-common-path lateral-shearing architecture, which optimizes the optical configuration for DHT in flow mode. Furthermore, we resolved particular computational issues necessary for the accurate retrieval of 3D R-I tomograms. Experimental tests were conducted on monocyte and mouse embryonic fibroblasts NIH-3T3 cells lines to validate the overall system for DHT operation, i.e., to validate the combination of optical hardware design and a new processing pipeline for tomographic reconstruction. Moreover, we found a new experimental strategy for testing the system’s reliability by assessing the repeatability of the DHT apparatus.

## 2. Materials and Methods

### 2.1. Experimental Setup

**Figure 1:**
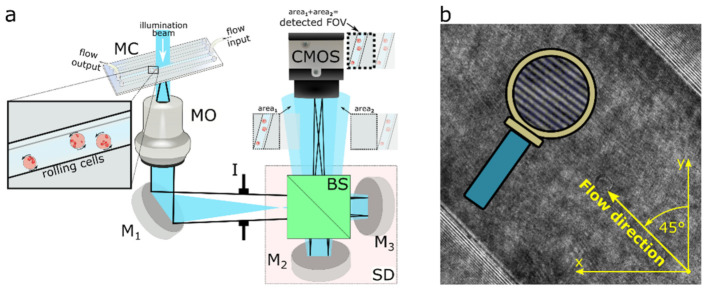
(**a**) Sketch of experimental arrangement. MO—microscope objective; MC—microfluidic chip; Ms—mirrors; I—iris diaphragm; BS—beam splitter; CMOS—camera; SD—shearing device. The image in front of the MO provides a zoom into the MC with cells rolling on the channel-side wall. Inserts in front of the CMOS illustrate replicas arising from the SD. The highlighted image portions (area_1_ and area_2_) represent overlapping areas detected by the CMOS, providing correct holographic performance. (**b**) Interference snapshot with a cell flowing inside the diagonally oriented (45 degrees) microfluidic channel. The zoom of the interference fringes is reported as inset within the draw of the magnifying glass.

Figure 1 shows a simplified sketch of the implemented laboratory setup where the spatially filtered and collimated laser beam (Sapphire SF, λ = 488 nm) illuminates the sample inside the microfluidic chip (MC, Straight 4-channel Mini-Luer Chip (P/N 10000091)). The MC is a part of the whole flow-cell unit (not fully sketched in Figure 1), consisting of the pressure controller (Elbeflow OB1), which is linked to the plastic syringe with the sample, thus serving as the input reservoir, the MC, the plastic outlet tube, and the waste glass. The regular sample flow is achieved by employing the controller in the constant-pressure mode. The flowing sample is subsequently imaged by a microscope objective (MO; Nikon, 20×/0.50) directly into the plane of the camera’s chip (CMOS; UI-3370CP-M-GL, 2048 × 2048, 5.5 μm square pixels). Here, the SD consists of a beam splitter (BS) and two mirrors M_2_ and M_3_; thus, two duplicated images of the studied sample are created. The first image arises from the optical path BS→M_2_→BS, and the second arises following the path BS→M_3_→BS. These two replicas are directed towards the camera with slightly different inclination angles and lateral displacements, due to the different tilts of the mirrors M_2_ and M_3_. Consequently, a portion of the observed field (Figure 1a; area_1_) serves as a signal beam, while the sample-free area in the replica (Figure 1a; area_2_) serves as a reference beam. If the coherence conditions of the interfering waves are satisfied, the interference fringes arise (Figure 1b), thus enabling single-shot off-axis holographic recordings and subsequent numerical reconstructions. In the present experimental configuration, the lateral shift among both replicated images is comparable to the dimensions of the used CMOS chip, so only one of the replicas is observed in a snapshot image. However, in principle, it is feasible to exploit both replicas to enhance the quality of the retrieved complex amplitude of the studied object, as was proposed in [47]. The Iris diaphragm (I), inserted between the MO and CMOS, reduces the amount of unwanted back-reflections and stray light. The lateral magnification of the imaging system, measured by a positive USAF 1951 amplitude line target, was established as 55, and the expected theoretical lateral resolution in the object space can be approximated as 0.82λ/NA ≈ 0.8 μm. In the proposed setup, the channel is tilted with respect to the camera; thus, the flow direction of the cell in Figure 1b (highlighted in yellow) is tilted 45 degrees with respect to the y axis of the introduced x–y reference system.

### 2.2. Sample Preparation

In our experiments, we use two cell lines: monocytes THP-1 and mouse embryonic fibroblasts NIH-3T3. THP-1 is a monocyte isolated from peripheral blood from an acute monocytic leukemia patient. This cell line can be used in immune-system-disorder research, immunology research, and toxicology research. The base medium for this cell line is ATCC-formulated RPMI-1640 Medium (Life technologies, ref 31870-025, Carlsbad, CA, USA). To make the complete growth medium, the following components are added to the base medium: fetal bovine serum to a final concentration of 10% (Life Technologies 10270), 2mM L-Glutamine (Lonza, Cat N.: BE17-605E, Basel, Switzerland), and 1% Penicillin/Streptomycin (Lonza, Cat N. DE17-602E). It is then maintained in cell culture flask (Corning, product number 353018, Corning, NY, USA) at 37 °C in a humidified atmosphere with 5% CO2. On the day of the experiment, they are harvested from the cell culture flask and transferred into a centrifuge tube containing 7.0 mL complete growth medium, and spun at approximately 125× *g* for 5 min; they are then resuspended in the complete medium and injected into the microfluidic channel at a final concentration of 3 × 105 cells/mL. Mouse embryonic fibroblasts NIH-3T3 are cultured in Dulbecco’s modified Eagle’s medium (DMEM), which contains 4.5 g L^−1^ D-glucose, and integrated with 10% fetal bovine serum (FBS) (Life Technologies, Carlsbad, CA, USA), 100 units per mL penicillin, and 100 μg mL^−1^ streptomycin (Sigma, St. Louis, MO, USA). They are harvested from the tissue culture flasks by incubation with a 0.05% trypsin–EDTA solution (Sigma, St. Louis, MO, USA) for 5 min, and centrifuged and resuspended in phosphate buffered saline (PBS). The final concentration is fixed to 2 × 105 cells per mL. Finally, the addition of 20 mM HEPES (Sigma-Aldrich, St. Louis, MO, USA) is made to provide extra buffering capacity, thus ensuring the right conditions for the cell culture medium during the manipulation outside the CO_2_ incubator.

### 2.3. Hologram Processing

**Figure 2:**
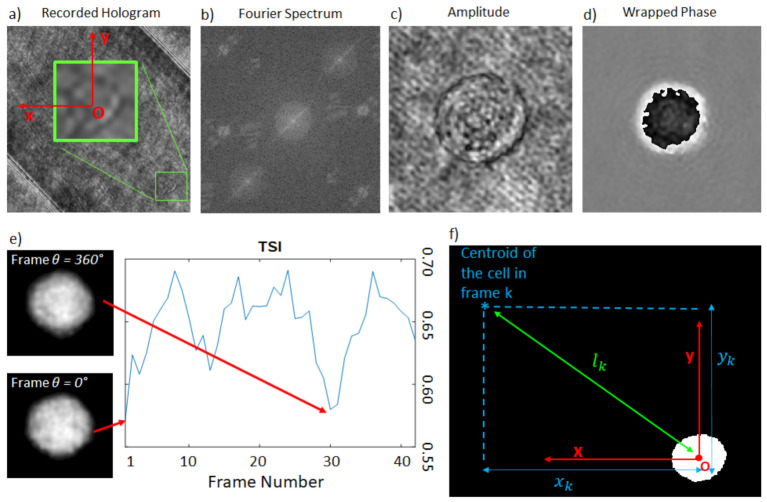
Steps of numerical processing. (**a**) recorded hologram, (**b**) Fourier spectrum, (**c**) reconstructed amplitude (**d**) reconstructed wrapped-phase distribution, (**e**) TSI used to individuate the θ = 360° phase map, including unwrapped phase images, (**f**) scheme illustrating the coordinate system and parameters for the rolling angle retrieval.

The recorded holograms are numerically processed to obtain in-focus complex amplitudes of the rolling cells. First, the selected interference record H (Figure 2a) is apodized using the tapered cosine function to reduce border effects, and subsequently Fourier transformed. The valuable diffraction order is extracted from the remaining spectral terms (Figure 2b), centered, and inverse-Fourier transformed. The retrieved complex amplitude, say $U\left(x,y,z\right)$, is further numerically propagated by the angular spectrum approach [48] up to the in-focus plane $z=\bar{z}$, which can be recovered by minimizing a suitable image sharpness metric. In our case, we employ the Tamura coefficient [49]:

(1) $$TC\left\{\left|U\right|\right\}\left(z\right)=\sqrt{\frac{\sigma_{xy}\left\{\left|U\right|\right\}\left(z\right)}{\mu_{xy}\left\{\left|U\right|\right\}\left(\left(z\right)\right)},}$$

where $\sigma_{xy}\left\{\left|U\right|\right\}\left(z\right)\;$and $\mu_{xy}\left\{\left|U\right|\right\}\left(\left(z\right)\right)$ are the standard deviation and the mean operators calculated over the amplitude $\left|U\right|$ along the $\left(x,y\right)$ variables, respectively, which are functions of the reconstruction depth z.

It should be noted that the object–plane distance $\bar{z}$ was established from the first frame containing the studied cell, and preserved for all subsequent holograms in the sequence. Moreover, the reference cell-free hologram $H_{ref}$ in the evaluated region was selected from the in-flow sequence and processed identically to H to remove optical aberrations and reduce the channel sharp edges, thus making cell tracking more comfortable. The tracking was performed following the cell’s centroid at every frame. A square area containing the cell at its center was selected, where the 2π-modulo phase map was unwrapped using the PUMA algorithm [50]. Subsequently, a denoising procedure, based on two-dimensional windowed Fourier transform filtering [51,52], was applied over the quantitative phase map (QPM) to attenuate the correlated speckle noise.

### 2.4. The Rolling Angle Recovery

To recover the projection angles of the cell, we applied the approach proposed in [27], but adapted for the 45-degree-tilted FoV. First, the frame corresponding to a full 360 degree rotation was found for all the QPMs, according to the metrics of the Tamura Similarity Index (TSI). The first QPM was associated to θ = 0 without loss of generality (Figure 2e), while the 360 frame is assigned as the one that minimizes the TSI.

Assuming the proportionality between the rotation and the translation motions, and considering the reference cartesian coordinates x,y centered over the centroid of the cell present in the first frame, the angular sequence was reconstructed according to the formula:

(2) $$\theta_{k}=0\;\Leftrightarrow \;k=1\;\mathrm{and}\;\theta_{k}=360\frac{l_{k}}{L_{360}}\;\Leftrightarrow \;k>1$$

where $\theta_{k}$ is the angle corresponding to the kth frame (Figure 2f) and $l_{k}$ is the distance between the centroids of the cell in the first and the kth frame. Due to the tilt configuration, $l_{k}$ is calculated as

(3) $$l_{k}=\sqrt{x_{k}{}^{2}+y_{k}{}^{2}},$$

where $x_{k}$ and $y_{k}\;$are the coordinates of the centroid at the kth frame in the introduced coordinate system, and $L_{360}$ is the same quantity as $l_{k}$ but specified for the frame where θ reaches 360°. Given the reduced framerate of the setup in use (i.e., 30 fps), an average of 35 projections is available from 0 to 360 degrees. Notice that, in the current microfluidic setting, the rotation velocity of the cells is not under control. This means that it is possible to miss the full rotation event, thus making a mistake in assigning the 360° frame. This scenario was already investigated in [27], in which it was demonstrated that, in similar microfluidic conditions and cell velocity ranges, the possible error in estimating the rotation angles provided negligible distortions in the reconstructed tomograms. Of course, in principle, it is possible to overcome this limitation by engineering the microfluidic module, allowing the behavior of cells’ motion to be predictable and precisely controlled. Then, a match between the frame rate and the velocity of the rotation can be set. The employed tomographic processing technique is discussed in the next section.

### 2.5. Reconstructions by Total Variation Minimization

Once the projection angles are retrieved, the tomogram can be accomplished employing a suitable retrieval algorithm. The filtered-back projection (FBP) is a standard inversion algorithm based on the inverse Radon transform, which is fast and exhibits low computational costs. However, when a reduced number of noisy projections are available from measurements, as happens for the set-up presented here, the inversion can perform poorly, introducing artifacts into the tomographic reconstruction. Various approaches can be considered to tackle this problem when only a few projections are available. One of them is to exploit the total variation minimization (TVM) approach [53]. TVM consists of minimizing a function, enforcing a regularized solution with an $l_{1}\;$penalty term applied to the so-called TV norm. TVM minimizes the following function:

(4) $$min\;\frac{\mu }{2}\|Ax-b{\|}_{2}{}^{2}+\|TV\left(x\right){\|}_{1},$$

where *A* represents the system matrix, *b* stands for the input data (the sinogram for the tomographic reconstruction case), x is the unknown to be searched for, $\frac{\mu }{2}$ represents the weighting factor, and *TV*(*x*) represents the TV norm of *x*, defined as $TV\left(x\right)=\nabla x$, with ∇ as the gradient operator.

The TV algorithm has become a popular algorithm thanks to its denoising and deblurring robustness, and its capability to preserve sharp edges in the function being reconstructed [54]. It is based on the piecewise constancy hypothesis of data, otherwise TV reconstructions can provide solutions with undesirable staircase behavior [55]. In the case of our area of interest, the subcellular structures may not satisfy the piecewise constancy hypothesis. In fact, in general, conventional TV reconstruction works efficiently in recovering the cell’s external geometry, but not the internal parts, thus forcing them into a piecewise constant map. In recent studies [56,57,58], approaches based on using TVM as a constraint within more sophisticated algorithms have allowed the hypothesis of piecewise constant function to be lifted. In other frameworks [59], the TV regularization is combined with a different tomographic solver (in the case mentioned, SART), where the derivative of the TV norm is used to adjust the estimated solution obtained via SART. Recently, to overcome the limitations of the conventional TVM method, high-order TV (HOTV) regularizers have been developed [54]. The idea behind HOTV approaches is to recover solutions balancing sharp edge discontinuities with but piecewise polynomial behaviors in the smooth regions. In the present case, we used the approach proposed in [60], where Polynomial Annihilation (PA) regularization [61,62] is utilized. At variance with classical TV, which encourages piecewise constant solutions, i.e., zeroth polynomials over the smooth regions, PA regularization promotes solutions of polynomial behavior of a prefixed degree in the smooth regions, allowing for a better retrieval of fine structures even for limited data, but with sparse boundary regions. The degree *k* of the searched polynomial is called the order of the HOTV. According to the PA regularization paradigm, Equation (4) is modified as:

(5) $$min\;\frac{\mu }{2}\;\|Ax-b{\|}_{2}^{2}+\|PA^{k}x{\|}_{1}$$

where $PA^{k}$ represents the k-order PA regularization term, which is computed as the *k*-derivative of the solution x along each of its dimensions. A formal mathematical description of PA regularization can be found in [60,62]. Note that, for *k* = 1, the PA reduces to the standard TV. In this paper, the solver used for the HOTV is that proposed by [63], based on the strategy developed in [64]. Furthermore, to reduce the search space, all of the reconstructions are performed under the non-negativity constraint [63].

### 2.6. Numerical Assessment of the Performance of HOTV

**Figure 3:**
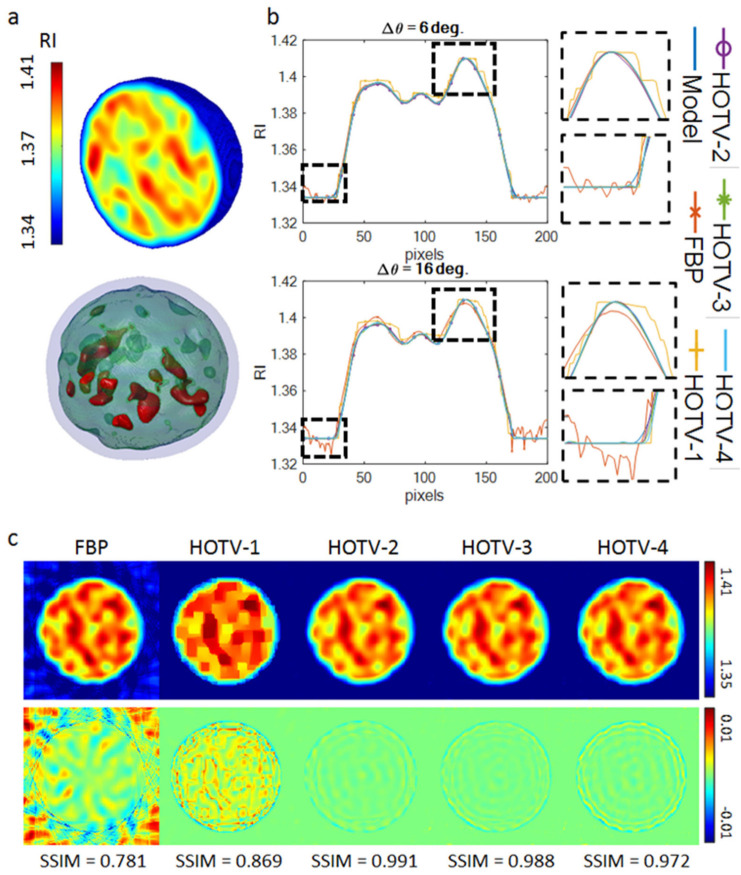
Monocyte simulation. (**a**) 3D (**top**) and isolevel (**bottom**) visualization of the simulated monocite, (**b**) horizontal cut through the central slice of the reconstructed tomograms for Δ*θ* = 6° and Δ*θ* = 16°, considering different solvers, (**c**) top row: central slice of the reconstructed tomograms considering different solvers for Δθ=16°, bottom row: difference slice between the reconstructions obtained with different solvers and the simulated model. Length of the reference scale bar is 5 µm.

In order to assess the performance of the considered regularization scheme, and to tune the parameters of the HOTV reconstruction, we developed a suitable numerical model of the cell. The model was created to provide a realistic tomogram as it would appear at the end of a tomographic reconstruction pipeline. Specifically, the RI distribution was simulated to have random Gaussian values within the range of [1.334 1.410], without defining recognizable internal structures with easy detectable RI distribution. The randomness of the RI values has the purpose of making the simulation more robust and more realistic, as compared to typical tomograms of suspended cells. Figure 3a illustrates the central slice and the isolevel visualization of a sample of the proposed model, representing the ground truth RI distribution. Regarding the isolevel visualization, two internal thresholds were employed, set as 0.33 and 0.66, respectively, of the simulated maximum RI value. The performance of the FBP and HOTV reconstructions were compared, considering HOTV of the first (HOTV-1), the second (HOTV-2), the third (HOTV-3) and the fourth (HOTV-4) order, respectively. Notice that HOTV-1 corresponds to the conventional TVM approach. The HOTV reconstruction method was controlled by several parameters [63] linked to the peculiar approach used for the minimization of Equation (5) [64]. Among others, important parameters are the data fidelity (DF), used to balance the data and the regularization terms; the order *k* of the HOTV; the number of iterations used in the minimization process, factorized in inner and outer loops; and the tolerances used to terminate the iterations. All the parameters available were left at their default settings, as reported in [63,64], except for the number of inner and outer iterations, which were both set equal to 30, and the DF parameter. The latter was tuned to optimize the performance in terms of the fidelity of the tomographic reconstruction with respect to the ground truth [65]. In our investigation, the optimal values of DF are DF = 24 for HOTV-1, DF = 48 for HOTV-2, DF = 96 for HOTV-3, and DF = 192 for HOTV-4. Moreover, all the reconstructions were performed under the non-negativity constraint, and the projection angles were in the range [0°, 360°]. The simulations were carried out considering the different angular steps $\Delta \theta =\theta_{k+1}-\theta_{k}{}_{}=6{}^{\circ}\;$(say, case A, Figure 3b) and Δθ=16° (say, case B, Figure 3c), corresponding to the mean and the maximum angular steps measured over realistic sequences, respectively. In particular, in case A, 60 projections contributed to the tomogram retrieval process while, in case B, just 22 projections were available.

In both of the considered cases, FBP suffered from noise, especially in the external regions of the retrieved tomogram (see bottom insets of Figure 3b,c). Unlike FBP, the HOTV solvers (Figure 3b,c) smooth the noise in the external region. Furthermore, in case B, FBP suffered from the reduced number of data, and did not recover the RI structures of the simulated cell accurately, as it is clearly observed in Figure 3c, bottom row. As expected, even HOTV-1 produced an unsatisfactory result, since it induces a piecewise constant behavior in the solution, not appropriate for describing the typical cell anatomy. On the contrary, the HOTV-2, -3, and -4 solvers performed successfully, since they were able to reconstruct the RI distributions quite accurately (Figure 3b,c), as expected. To compare the performance of the considered solvers, the structural similarity index (SSIM) between the ground truth and the reconstruction method was calculated over the entire tomogram. The FBP reaches SSIMFBP = 0.7808, the HOTV-1 reaches SSIMHOTV-1 = 0.8694, the HOTV-2 reaches SSIMHOTV-2 = 0.9908, the HOTV-3 reaches SSIMHOTV-3 = 0.9884, and the HOTV-4 reaches SSIMHOTV-4 = 0.9724. These results confirm the better accuracy of HOTV-2 and HOTV-3 with respect to both HOTV-1 and FBP, showing slightly better results of HOTV-2 against HOTV-3. HOTV-4 reconstructions appear slightly less accurate due to some artifacts, resulting from the wider variability of searched-for unknowns. HOTV-2 is selected as it definitively had the best performance among the numerically analyzed HOTV algorithms.

## 3. Results

**Figure 4:**
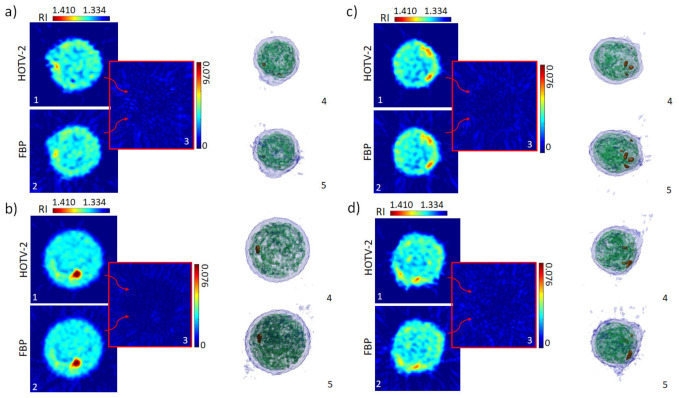
Tomograms of the four different monocytes examined. For every panel (**a**–**d**), cross-section slices from the HOTV-2 tomographic reconstruction (subfigures **a1**–**d1**), cross-section slices from the FBP tomographic reconstruction (subfigures **a2**–**d2**), and the absolute value of the difference between the two cross-section slices (subfigures **a3**–**d3**) are presented, together with isolevel visualizations for the HOTV-2 (subfigures **a4**–**d4**) and FBP reconstructions (subfigures **a5**–**d5**). The length of the reference scale bar is 5 µm.

The obtained QPMs and the retrieved angular sequence were used as the input parameters of both the gold-standard FBP and HOTV tomographic reconstruction algorithms, respectively. Specifically, the FBP and HOTV-2 approaches were employed for the tomogram reconstruction using QPMs corresponding to the cell orientation range from 0° to 360°. Notice that, in order to correctly use the implemented code for both methods, the QPM was rotated 45 degrees to align the flow direction along the *y*-axis, and the ultimate square area of 20 × 20 μm^2^ containing the cell in the center was established. This procedure was repeated for all of the images in the sequence of the studied single cell. The reconstructions obtained with both algorithms are reported in Figure 4, comparing the results obtained to the real data. First, tomograms of four different monocytes, observed rolling in the diagonally tilted microfluidic channel, were reconstructed.

**Figure 5:**
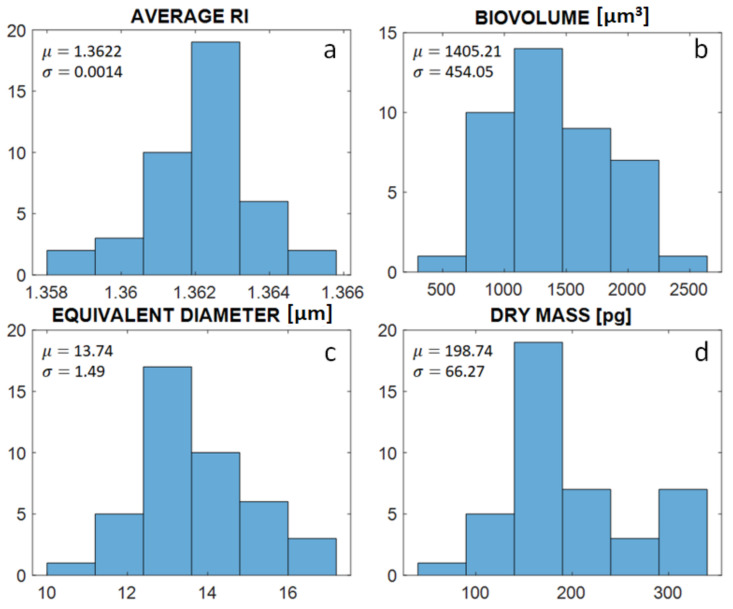
Histograms computed over the monocytes under examination; (**a**) average refractive index, (**b**) biovolume, (**c**) equivalent diameter, (**d**) dry mass.

Cross-section slices of the tomograms obtained using HOTV-2 are reported in Figure 4a1–d1. In contrast, the same slices reconstructed using FBP are presented in Figure 4a2–d2. In the HOTV-2 reconstructions, a homogeneous reconstructed background can be observed which is opposite to that seen for FBP, as was previously discussed for the numerical simulations (Figure 3). Additionally, HOTV-2 seems to exhibit higher RI contrast than the FBP, which provides smoother RI profiles. This beneficial effect of the HOTV-2 algorithm compared to the FBP is clearly visible when considering the absolute difference between the two cross-section slices (Figure 4(a3–d3)), in which the ability of the HOTV-2 to reduce the artifacts introduced by the FBP in both the internal region of the cell and the background is highlighted. Insights into the cells’ anatomy are visualized in the isolevel plots for both algorithms, HOTV-2 (Figure 4(a4–d4)) and FBP (Figure 4(a5–d5)). Finally, Figure 5 shows histograms of conventional morphological features, such as the average RI (Figure 5a), the biovolume (Figure 5b), the equivalent diameter (Figure 5c), and the dry mass (Figure 5d), evaluated over the entire number of monocytes examined, in the case of HOTV-2 reconstructions. These features have been demonstrated to be the most informative ones for label-free single-cell analysis, encoding the measurements of cells cycles and becoming decisive in cell phenotyping and disease identification [66,67]. We also evaluate the mean value μ and the standard variation σ of such histograms, finding that they are comparable with the expected tabular ones reported in other studies [68,69].

**Figure 6:**
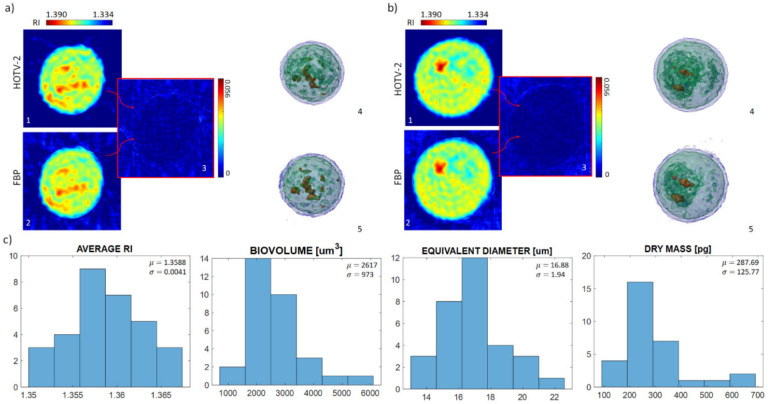
Tomograms of two NIH-3T3 cells. For both panels (**a**,**b**), cross-section slices from the HOTV-2 tomographic reconstruction (subfigures **a1**,**b1**), cross-section slices from the FBP tomographic reconstruction (subfigures **a2**,**b2**), and the absolute value of the difference between the two cross-section slices (subfigures **a3**,**b3**) are presented, together with isolevel visualizations for the HOTV-2 (subfigures **a4**,**b4**) and FBP reconstructions (subfigures **a5**,**b5**). (**c**) Histograms of average refractive index, biovolume, equivalent diameter, and dry mass, computed over the NIH-3T3 under examination.

To further validate the proposed approach, the presented analysis was also conducted on a second cell line, namely mouse embryonic fibroblasts NIH-3T3. Figure 6 reports the results obtained in this case. Figure 6a,b and relative subfigures present the details of the reconstructions of two cells. As in the first case, FBP and HOTV-2 solvers were considered, with the latter showing the advantages previously discussed. Figure 6c reports the histograms relative to the same morphological parameters evaluated in Figure 5, calculated over the entire population of examined NIH-3T3 cells. The obtained values fall into the expected range according to literature [26].

**Figure 7:**
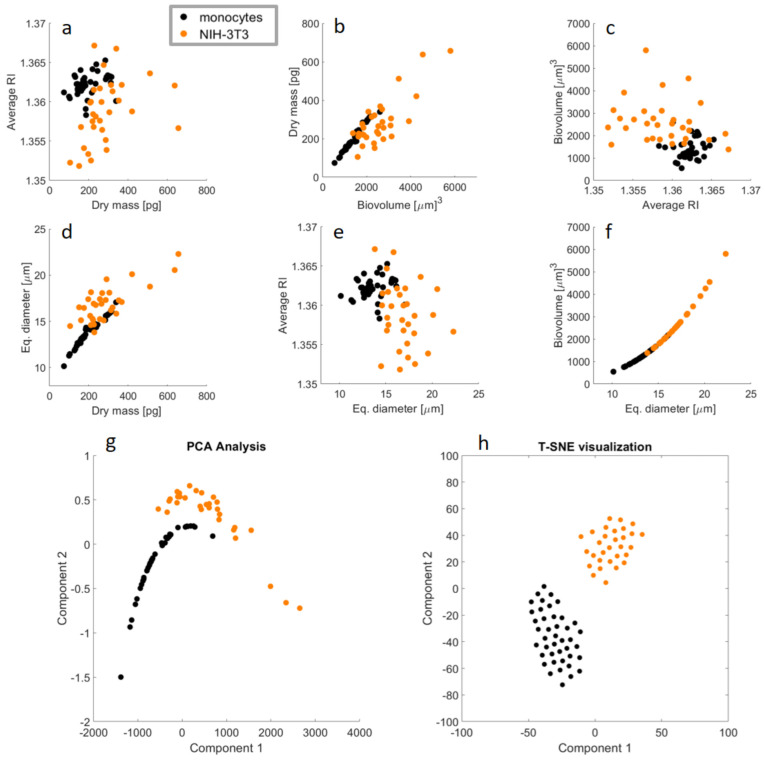
Data analysis comparing monocytes and NIH-3T3 cell lines; (**a**–**f**) display the scatterplots between each pair of the conventional morphology features calculated for the analyzed cell lines, i.e., average RI, dry mass, biovolume, and equivalent diameter. In particular, the subfigure (**f**) shows a quasi-linear trend, due to the obvious correlation between biovolume and equivalent diameter. This renders redundant the information content provided by the pair of subfigures (**b**) and (**d**) as well as (**c**) and (**e**), which display similar population distributions. Reported in (**g**,**h**) are two of the most popular methods for inspecting high-dimensional data, i.e. PCA analysis and the T-SNE visualization, respectively; these have the effect of removing such redundancy and show the spatial separation of the two analyzed cell populations, thus demonstrating the possibility of clustering them.

The morphological features reported in Figure 5 and Figure 6 can be used to visualize the main differences between the two analyzed cell lines. Therefore, as a direct comparison between them, Figure 7a–f show the scatterplots among the four features calculated above. Notice that the biovolume and the equivalent diameter are strongly correlated with each other, as highlighted by the quasi-linear trend of the scatterplot in Figure 7f. This means that the pair of scatterplots in Figure 7b,d, as well as in Figure 7c,e, represent similar population distributions. It is evident that the NIH-3T3 cell line shows a wider morphological variability compared to monocytes, as shown by the more widely spread values in the scatterplots. This corresponds to a wide heterogeneity within the NIH-3T3 population. In order to investigate the possibility of performing data clustering, we report in Figure 7g,h the two most-used methods for inspecting high-dimensional data, i.e., principal component analysis (PCA) and t-distributed stochastic neighbor embedding (T-SNE) visualization. Even if the clustering of the two analyzed cell populations appears feasible using only the four the morphological features calculated here, as clearly visualized in Figure 7g,h, the accurate classification of different cells would require deeper tomographic data analysis, mainly focused on the engineering and selection of advanced 3D image features, and/or suitable artificial neural networks.

### Repeatability of the Experimental Reconstruction Process

In our experimental setting, there is also the possibility of imaging the same cell multiple times. In fact, the flow cell unit used here (see ‘Experimental setup’) enables fluent pressure settings inside the microfluidic chip; thus, the speed of the in-flow cells is driven in a controlled way. If the flow unit apparatus is appropriately adjusted, the cell-rolling direction can be reversed, and both forward and backward flow directions are achieved by changing the pump pressure. In this way, the cell was moved into its initial position and observed repeatedly, considering the same flow direction.

**Figure 8:**
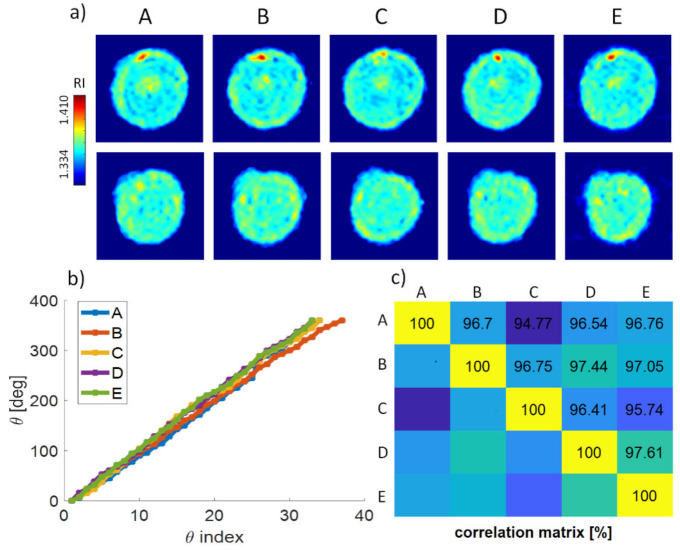
(**a**–**c**) Repeatability of the experimental reconstruction process. (**a**) Comparison between reconstructions of the same tomograms for five different observations of the same cell [A–E] which is rotated five times through the channel: central (top row) and peripheral (bottom row) x-z slices of the tomograms obtained by HOTV-2; (**b**) retrieved angular sequences for the five considered experiments; (**c**) correlation matrix calculated between the tomograms reconstructed for the five considered independent observations.

Subsequently, rolling angles for five various observations of the same monocyte cell were calculated, leading to the five independent tomographic reconstructions (Figure 8a,b). Cross-sections through the tomograms obtained by employing HOTV-2 are reported in Figure 7a, where the top row represents the central x-z slice for every occurrence: the same internal structure is observed. The bottom row represents a different x-z slice, in which a peculiar feature of the external shape of the reconstructed cell is highlighted. It should be noted that similarity among individual observations may be reduced by effects influencing the cell anatomy, e.g., cell deformation by hydrodynamic forces or adhesion to the channel [70]. Nevertheless, the repeatability of the measurements was established using the correlation coefficient as a similarity measure among all five individual tomograms, reaching a mean value of 96.6%. The established correlation matrix (Figure 8c) reports a good accordance for all values, higher than ≈95%, thus indicating the robustness of the experimental recordings and the numerical processing. Finally, the correlation coefficient was calculated between the tomogram of Figure 8a in the case of column A and the tomograms present in Figure 4, obtaining a mean value of 75.53%, thus referencing all the above calculations.

## 4. Discussion and Conclusions

In this work, we reported a complete and thorough investigation into a DHT system suitable for in-flow tomographic cytometry. The apparatus was realized using a common-path lateral-shearing digital holographic microscope with conventional components—a method proposed here for the first time. Experimental measurements were acquired using a relative low-resolution microscope objective (NA = 0.5) and an ordinary CMOS camera, thus reducing the financial costs incurred by the equipment. Such reduced optical performance allowed us to make a further step towards the setup of a Lab-on-Chip device, but posed several limitations, making the tomographic reconstructions challenging. Particular care was paid to dealing with the reduced frame rate of conventional cameras, which provide a few tens of projections for the considered system, while still ensuring constant flow conditions. Hence, an unconventional approach with a diagonally tilted microfluidic channel was adopted, allowing the detected path to be elongated for the established experimental arrangement, which allowed us to measure more projections for cells flowing close to the channel’s center, regardless of cells flowing close to the periphery. The experimental investigation was performed by considering monocyte and mouse embryonic fibroblast NIH-3T3 cells lines, which were reconstructed using numerical algorithms suitable for reduced datasets. The high-order total variation approach was applied to in-flow tomographic cytometry for the first time, and its superiority with respect to the standard filtered back projection and first-order total variation approaches was demonstrated, employing a numerically modeled cell as a ground truth. Moreover, various living cells were recovered with their characteristics, including an average refractive index, biovolume, equivalent diameter, and dry mass, achieving results that concur with the tabular values. In addition, the repeatability of the overall experimental numerical performance was proved by independent observations of the same monocyte cell, guided by the controlled in-flow conditions. The retrieval robustness was quantified using the correlation coefficient as a metric, which found ~97% similarity among five independent observations, proving the consistency of the overall tomographic retrieval process. Overall, the achieved results show that an optimized configuration of both optics and the computational key aspects could be used for a DHT in-flow cytometry model for lab-on-chip and label-free biomedical applications in the future.

## Data Availability

Not applicable.
